# ﻿Taxonomic review of the genus *Ammathella* Volynkin, 2019 with descriptions of three new species from southeastern Xizang, China (Lepidoptera, Erebidae, Arctiinae, Lithosiini)

**DOI:** 10.3897/zookeys.1114.86182

**Published:** 2022-07-27

**Authors:** Xiong-Yan Yin, Anton V. Volynkin, Yu-Long Zhang, En-Yong Chen, Si-Yao Huang, Zhao-Hui Pan

**Affiliations:** 1 College of Plant Science, Tibet Agricultural and Animal Husbandry University, Linzhi 860000, China; 2 Altai State University, Lenina Avenue, 61, RF-656049, Barnaul, Russia; 3 Department of Entomology, College of Plant Protection, South China Agricultural University, Guangzhou 510642, Guangdong, China; 4 Key Laboratory of Forest Ecology in Tibet Plateau (Institute of Plateau Ecology, Tibet Agricultural and Animal Husbandry University), Ministry of Education, Linzhi 860000, China

**Keywords:** *Asura/Miltochrista* complex, Himalaya, India, lichen moth, Myanmar, Nudariina, taxonomy

## Abstract

A brief taxonomic review of the lithosiinid genus *Ammathella* Volynkin, 2019 is presented. Three new species of the genus *Ammathella* Volynkin, 2019 are described from Motuo County, southeast Xizang: *A.longicornuta* S.-Y. Huang, Yin & Volynkin, **sp. nov.**, *A.lhoba* S.-Y. Huang, Yin & Volynkin, **sp. nov.**, and *A.monpa* S.-Y. Huang, Yin & Volynkin, **sp. nov.** A key to the species of the genus is provided. Adults and genitalia of both sexes are illustrated.

## ﻿Introduction

The genus *Ammathella* Volynkin, 2019 (type species: *Barsinegaro* Volynkin, 2018) is a compact lichen moth genus distributed mainly in eastern part of the Himalaya and currently comprising four species: *Ammathellagaro* (Volynkin, 2018) from Assam, northeastern India, *A.midzhan* (Volynkin, 2018) and *A.shingwa* Volynkin & S.-Y. Huang, 2020, both from Kachin, northeastern Myanmar, and *A.gesar* Huang & Volynkin, 2020 from Xizang, southwestern China. Originally *Ammathella* was described as a subgenus of the genus *Ammatho* Walker, 1855 (Volynkin et al. 2019) to include two species formerly described in the genus *Barsine* Walker, 1854 (Volynkin 2018), but subsequently it was upgraded to generic level by Huang et al. (2021) based on molecular and morphology evidence.

*Ammathellagesar* is the only member of the genus currently known to the Chinese fauna, and its type locality is Pailong Village located at the northern slope of the Himalaya in Xizang. However, in the course of identifying the Lithosiini housed in the Tibet Agricultural and Animal Husbandry University, was found a series of specimens of *Ammathella* from Beibeng and Bononggong (also well-known as 80K) villages in Motuo County, located at the southern slope of the Himalaya. The examination of the male and female genitalia of these individuals revealed that they belong to three species new to science, which are described below. Based on our past studies and new discoveries, the genus is briefly reviewed and a key to the genus is also provided.

## ﻿Materials and methods

The specimens examined are deposited in the following institutional collections: Tibet Agricultural and Animal Husbandry University (**TAAHU**), Linzhi, Xizang, China; South China Agricultural University (**SCAU**), Guangzhou, China, and Museum Witt Munich in the the Bavarian State Collection of Zoology (Museum Witt München / Zoologische Staatssammlung, München, **MWM/ZSM**), Munich, Germany. The moths were collected at night using light traps. Photographs of the adults of the new species were taken using a Sony DSC-RX100 v1.00 camera, a Canon 5DIII camera, and a Nikon D3100 camera with AF-S Nikkor 18–55 mm lens. The abdomens of the specimens were removed and macerated in 10% NaOH or 8% KOH solution for the examination of genitalia. Photographs of the genitalia of *Ammathellagesar* were taken under a Keyence VHX-5000 digital microscope. The genitalia of *A.longicornuta*, *A.lhoba*, and *A.monpa* were imaged using a Nikon SMZ18 photograph system, and those of other taxa were taken by a Nikon D3100 camera with AF-S Nikkor 18–55 mm lens attached to a microscope with an LM-scope adapter. All pictures were processed using Adobe Photoshop CS5 software. The terminology for adults and genitalia morphology follows Volynkin et al. (2019) and Huang et al. (2020).

## ﻿Taxonomy

### ﻿Genus Ammathella

Taxon classificationAnimaliaLepidopteraErebidae

Volynkin, 2019

8A49E3DD-4472-5505-8743-376DE7D4C106

Ammatho (Ammathella) Volynkin in Volynkin, Huang & Ivanova 2019, Ecologica Montenegrina 26: 19, figs 7, 8, 107, 163.

#### Type species.

*Barsinegaro* Volynkin, 2018 [type locality: Garo Hills, Assam, India], by original designation.

#### Diagnosis.

Externally, species of the genus display a wing pattern typical of members of the *Asura*/*Miltochrista* generic complex and consisting of a dull yellowish ground color of the forewing with reddish lengthwise streaks and blackish transverse lines, reminiscent of the genera *Sarbine*, *Processine*, *Ammatho*, and *Moorasura* Volynkin & Huang, 2019. In male genitalia, *Ammathella* is characterized by the narrow and elongated valva, the weakly sclerotized medial costal process which is short, swollen and strongly broadened basally, and the small and apically rounded distal costal process. The phallus vesica of *Ammathella* is rather unique within the genus in bearing a cluster of strong and short cornuti on its ventral side. The female genitalia of *Ammathella* are characterized by the densely spinulose corpus bursae and the relatively short and sclerotized appendix bursae positioned postero-ventrally and directed posteriorly (Volynkin et al. 2019; Huang et al. 2020).

#### Description.

**External morphology of adults**. Forewing length 7.5–14 mm in males and 9.5–14 mm in females. Antenna long, weakly ciliate in both sexes. Sexual dimorphism limited: female with broader forewing and paler abdomen. Head and thorax coloration varying from pale orange to reddish-orange, tegula with blackish spot medially. Forewing broad, triangular with rounded apex. Forewing ground color varying from pale orange to orange-red with yellow streaks on veins and yellow irregular spot in discal area; markings dark brown. Costal margin edged with black scales. Basal spot small and round. Antemedial area with dark brown streak on costa (except *A.shingwa*), in certain species with two spots subbasally. Transverse lines dilated medially and anteriorly. Antemedial line smoothly convex outwards anterio-medially. Medial line sinuous, X-like fused with antemedial line in cell. Medial area between medial and postmedial lines with irregular spot in cell. Postmedial line smoothly convex outwards anterio-medially, in most species touching medial line at costa. Veins in postmedial area with lengthwise dark brown streaks and intense yellow suffusion. Terminal line blackish-brown, thin with short triangular denticles on veins. Cilia blackish-brown. Hindwing ground color pale pinkish-red or ochreous, paler subbasally and deeper or with blackish suffusion subapically. Cilia blackish around apex and pinkish or ochreous, scattered with black medially and at tornus. Abdomen covered with pale pinkish or ochreous hair-like scales, in males, with admixture of blackish scales in distal half or third.

**Genitalia. Male.** Uncus long and slender, smoothly down curved, distally tapered and apically pointed. Tuba analis moderately broad (ca 1/3 of tegumen length) with thin and weakly sclerotised scaphium and setose subscaphium. Tegumen triangular, moderately sclerotised. Vinculum shorter than tegumen, robust, V- or U-shaped. Valva elongate and relatively narrow. Costa with swollen and apically rounded medial process (vestigial in *A.longicornuta* sp. nov.) and narrow and apically rounded distal process (broad in *A.longicornuta* sp. nov.). Membranous lobe of valva large and broad. Sacculus well-sclerotized, dorsally setose in certain species. Distal saccular process short and narrow, rounded or pointed apically, up curved (but directed distally in *A.longicornuta* sp. nov.). Juxta broad, plate-like with medial membranous commissure, in certain species also with apical process. Phallus moderately broad, tubular, more or less straight or in a somewhat S-like curve. Vesica broad with large sack-like or conical subbasal diverticulum in certain species bearing small cluster of cornuti distally or laterally. Medial diverticulum large, semiglobular, bearing broad cluster of numerous short cornuti. Ventro-lateral medial diverticulum with cluster of small and short cornuti of various sizes and shapes. Ventral diverticulum elongate, covered with numerous small cornuti or spinules. Basal plate of vesica ejaculatorius short and narrow. **Female.** Papilla analis trapezoid with rounded corners. Apophyses long and thin, more or less equal in length. Ostium broad. Antrum broad and heavily sclerotized, in certain species bearing subostial ligula. Ductus bursae broad and very short, with sclerotized posterior and membranous anterior sections. Corpus bursae large, sack-like, densely covered with numerous spinules medially and posteriorly. Anterior section of corpus bursae densely covered with spinulose scobination, with round or elliptical signum (absent in *A.longicornuta* sp. nov.). Appendix bursae small, sclerotized, elliptical or conical, positioned postero-ventrally.

#### Distribution.

Species of the genus are known from northeast India, northern Myanmar and southwestern China (Xizang) (Volynkin 2018; Volynkin et al. 2019; Huang et al. 2020).

##### ﻿Species content of the genus *Ammathella*

– *A.longicornuta* S.-Y. Huang, Yin & Volynkin sp. nov. (China: SE Xizang)

– *A.lhoba* S.-Y. Huang, Yin & Volynkin sp. nov. (China: SE Xizang)

– *A.monpa* S.-Y. Huang, Yin & Volynkin sp. nov. (China: SE Xizang)

– *A.gesar* S.-Y. Huang & Volynkin, 2020 (China: SE Xizang)

– *A.garo* (Volynkin, 2018) (NE India: Assam)

– *A.midzhan* (Volynkin, 2018) (N Myanmar: Kachin State)

– *A.shingwa* Volynkin & S.-Y. Huang, 2020 (N Myanmar: Kachin State)

### ﻿ Ammathella longicornuta

Taxon classificationAnimaliaLepidopteraErebidae

S.-Y. Huang, Yin & Volynkin sp. nov.

18AA073C-A3FA-5ADA-8D86-E357AEBE631B

https://zoobank.org/E4DF9179-D9C9-4159-981E-439D893EA933

**Figure 1–10.**
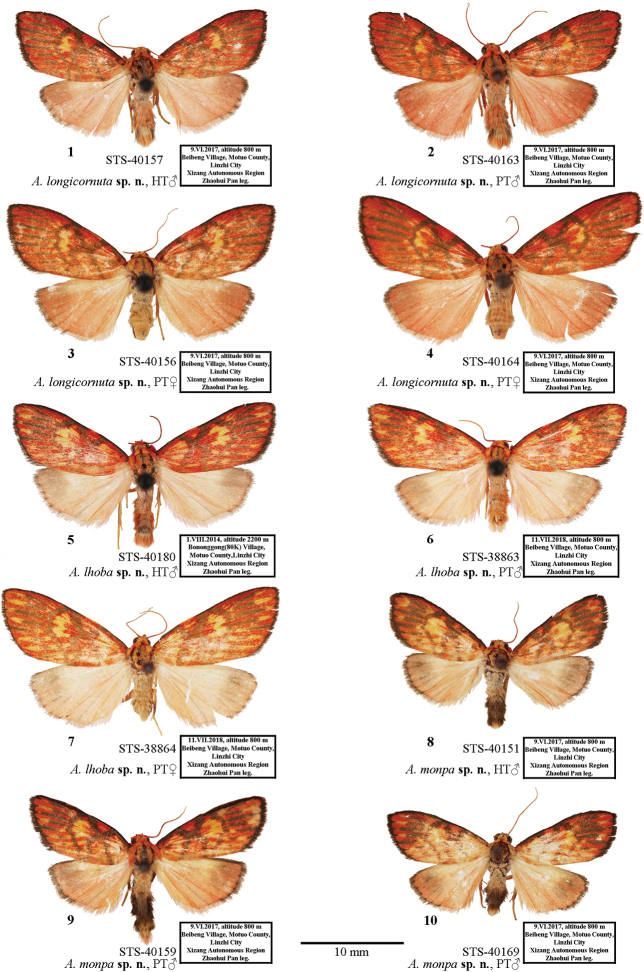
Adults of *Ammathella***1** holotype of *A.longicornuta* sp. nov., male, STS-40157, SW China (TAAHU) **2** paratype of *A.longicornuta* sp. nov., male, STS-40163, SW China (TAAHU) **3** paratype of *A.longicornuta* sp. nov., female, STS-40156, SW China (TAAHU) **4** paratype of *A.longicornuta* sp. nov., female, STS-40164, SW China (TAAHU) **5** holotype of *A.lhoba* sp. nov., male, STS-40180, SW China (TAAHU) **6** paratype of *A.lhoba* sp. nov., male, STS-38863, SW China (TAAHU) **7** paratype of *A.lhoba* sp. nov., female, STS-38864, SW China (TAAHU) **8** holotype of *A.monpa* sp. nov., male, STS-40151, SW China (TAAHU) **9** paratype of *A.monpa* sp. nov., male, STS-40159, SW China (TAAHU) **10** paratype of *A.monpa* sp. nov., male, STS-40169, SW China (TAAHU).

Figs 1–4 , 19 , 20 , 31

#### Type material.

***Holotype***: male, altitude 800 m, 9.VI.2017, Beibeng Village, Motuo County, Linzhi City, Xizang Autonomous Region, China, Zhao-hui Pan leg., slide STS-40157 (TAAHU). ***Paratypes***: 5 males, 3 females, same data as the holotype, slides STS-40148, STS-40154, STS-40155 & STS-40163 (male); STS-40156, STS-40162 & STS-40164 (female) (TAAHU).

#### Diagnosis.

**Figure 11–18.**
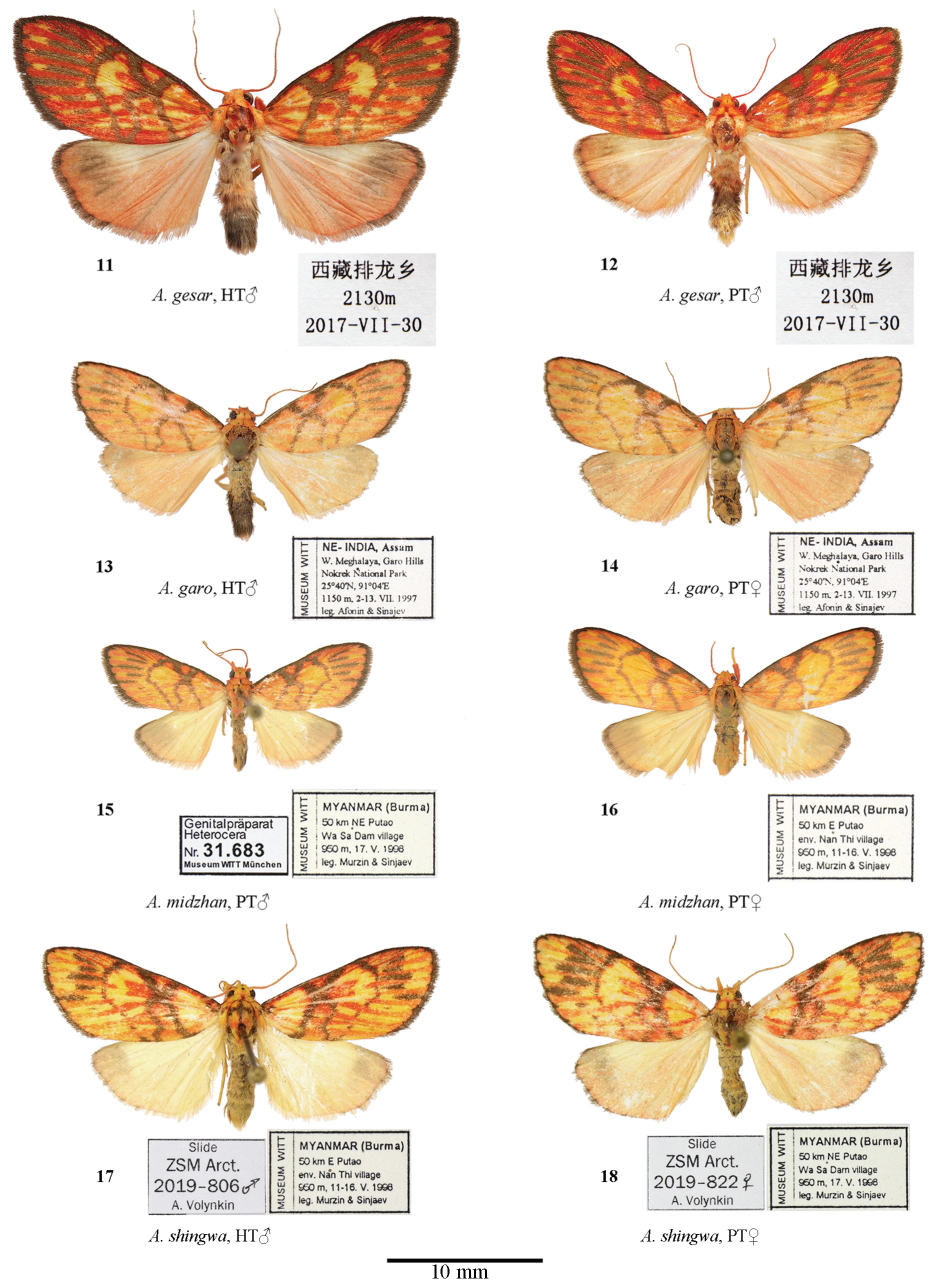
Adults of *Ammathella***11** holotype of *A.gesar*, male, SW China (SCAU) **12** paratype of *A.gesar*, male, SW China (SCAU) **13** holotype of *A.garo*, male, MWM 31769, NE India (MWM/ZSM) **14** paratype of *A.garo*, female, MWM 31770, NE India (MWM/ZSM) **15** holotype of *A.midzhan*, male, MWM 31683, N Myanmar (MWM/ZSM) **16** paratype of *A.midzhan*, female, MWM 31768, N Myanmar (MWM/ZSM) **17** holotype of *A.shingwa*, male, ZSM Arct. 2019-806 Volynkin, N Myanmar (MWM/ZSM) **18** paratype of *A.shingwa*, male, ZSM Arct. 2019-822 Volynkin, N Myanmar (MWM/ZSM).

Length of forewing 11.4–11.9 mm (*n* = 5, 11.5 mm in holotype) in males and 12.6–13.1 mm (*n* = 3) in females. *Ammathellalongicornuta* sp. nov. is externally reminiscent of *A.gesar* (Figs 11, 12, 25, 26) and *A.garo* (Figs 13, 14, 27, 33) but is distinguished by the male tip of abdomen, which is pinkish and covered with much lesser blackish hair-like scales than in the similar congeners, and the obsolete submarginal dull yellowish spots on the forewing (whereas they are prominent in *A.gesar* and *A.garo*). In the male genitalia, *A.longicornuta* sp. nov. is distinguished from the two similar congeners by the combination of the following characters. (1) The juxta lacks a medial process, similar to *A.gesar*, while the juxta of *A.garo* bears a long conical process. (2) The medial costal process is minute while it is large and prominent in *A.gesar* and *A.garo*. (3) The distal costal process is situated more distally than in *A.gesar* and *A.garo*, and is much broader and shorter, whereas it is lobe-like and markedly narrower in the congeners. (4) The distal saccular process is directed distally whereas it is up curved in *A.gesar* and *A.garo*. (5) In the phallus vesica, the subbasal diverticulum bears a small cluster of cornuti apically (absent in *A.gesar* and *A.garo*). (6) The semiglobular medial diverticulum bears a broad cluster consisting of several large and long cornuti basally and numerous smaller cornuti ventro-distally, while in *A.gesar* and *A.garo*, the cornuti in the semiglobular medial diverticulum are more or less equal in size. (7) The ventro-basal elongate diverticulum of *A.longicornuta* sp. nov. is longer than in *A.garo*, while it is absent in *A.gesar*. Since the female of *A.gesar* is unknown, the female genitalia of *A.longicornuta* sp. nov. are compared only to *A.garo*, from which the new species differs in the shorter and broader ductus bursae, and the lack of a signum bursae, which is present in *A.garo*.

#### Etymology.

The specific epithet *longicornuta* refers to the presence of long and large cornuti on the surface of the semiglobular medial diverticulum in vesica of male genitalia.

#### Distribution.

Currently known only from the type locality in Motuo County, southeastern Xizang, China.

### ﻿ Ammathella lhoba

Taxon classificationAnimaliaLepidopteraErebidae

S.-Y. Huang, Yin & Volynkin sp. nov.

964C57AA-6978-5ABA-A14F-C2D16FA4272C

https://zoobank.org/DF85F92B-D74A-4481-A306-A6E874F8D3A2

Figs 5–7 , 21 , 22 , 32

#### Type material.

***Holotype***: male, altitude 2200 m, 1.VIII.2014, 80K (Bononggong) Village, Motuo County, Linzhi City, Xizang Autonomous Region, China, Zhao-hui Pan leg., slide STS-40180 (TAAHU). ***Paratypes***: 2 males, 1 female, altitude 800 m, 11.VII.2018, Beibeng Village, Motuo County, Linzhi City, Xizang Autonomous Region, China, Zhao-hui Pan leg., slide STS-38860, STS-38863 (male); STS-38861, STS-38864 (female) (TAAHU).

#### Diagnosis.

Length of forewing 12.2–12.8 mm (*n* = 3, 12.6 mm in holotype) in males and 13.8–14 mm (*n* = 2) in females. Externally, *Ammathellalhoba* sp. nov. is similar to *A.gesar* (Figs 11, 12, 25, 26) and *A.midzhan* (Figs 15, 16, 28, 29, 34) but is distinguished by the tip of the male abdomen with a markedly sparser admixture of blackish hair-like scales. Additionally, compared to *A.midzhan*, the new species is significantly larger in size (the forewing length is 12.2–12.8 mm in male and 13.8–14 mm in female vs 7.5–8 and 9.5 mm respectively in *A.midzhan*). In the male genitalia, *A.lhoba* sp. nov. is more similar to *A.midzhan* than to *A.gesar* by sharing the similar phallus and vesica structure. However, *A.lhoba* sp. nov. differs from *A.midzhan* in the combination of the following characters. (1) The medial costal process is slightly shorter and smaller. (2) The cluster of cornuti on the subbasal diverticulum is larger and bearing granulation basally, while it is smaller and lacks granulation in *A.midzhan*. (3) The cluster of cornuti on the semiglobular medial diverticulum is larger and the cornuti are smaller and shorter. (4) The ventral medial diverticulum is covered with more cornuti of smaller size while in *A.midzhan*, while those cornuti are fewer and larger. In the female genitalia, *A.lhoba* sp. nov. differs from *A.midzhan* in the anterior section of the corpus bursae densely covered by spinules whereas it is thoroughly membranous in the congener.

#### Etymology.

The specific epithet *lhoba* refers to the local Lhoba people in Motuo County.

#### Distribution.

Currently known from two localities in Motuo County, southeastern Xizang, China.

### ﻿ Ammathella monpa

Taxon classificationAnimaliaLepidopteraErebidae

S.-Y. Huang, Yin & Volynkin sp. nov.

D28BBE77-0403-584C-8ABA-27311826BE16

https://zoobank.org/261A5113-6F0B-4039-B8D7-95943DBF0166

Figs 8–10 , 23 , 24

#### Type material.

***Holotype***: male, altitude 800 m, 9.VI.2017, Beibeng Village, Motuo County, Linzhi City, Xizang Autonomous Region, China, Zhao-hui Pan leg., slide STS-40151 (TAAHU). ***Paratypes***: 3 males, the same data as in the holotype, slides STS-40159, STS-40167 & STS-40169 (TAAHU).

#### Diagnosis.

Length of forewing 10.6–11.8 mm (*n* = 4, 10.6 mm in holotype). *Ammathellamonpa* sp. nov. is externally very similar to *A.gesar* (Figs 11, 12, 25, 26) and a reliable identification requires the examination of the genitalia structures. Compared to another similar species, *A.garo* (Figs 13, 14, 27, 33), the new species has hindwing cilia black from apex to vein CuA_2_, while it is pinkish, scattered with black, from apex to tornus in *A.garo*. In the male genitalia, *A.monpa* sp. nov. is distinguished from the two similar congeners by the combination of the following characters. (1) The juxta is much narrower and smaller. (2) The medial process of juxta is short, round, and covered with spinules thoroughly, whereas the juxta of *A.garo* bears a long conical medial process, and that of *A.gesar* lacks a process. (3) The distal costal process is situated more distally from the medial costal process, similar to that in *A.garo*, while it is situated closer to the medial costal process in *A.gesar*. (4) The distal costal process is longer than in *A.gesar* and *A.garo*. (5) The distal saccular process is apically rounded, similar to *A.garo*, while it is apically pointed in *A.gesar*. (6) The phallus is slender and straight, similar to *A.gesar*, while forms an S-like curve in *A.garo*. (7) In the phallus vesica of *A.monpa* sp. nov., the subbasal diverticulum is larger than in *A.gesar* and *A.garo*. Additionally, the cornuti on the ventral medial diverticulum of the new species are larger than in *A.gesar* and more similar to those in *A.garo*.

**Figure 19–22.**
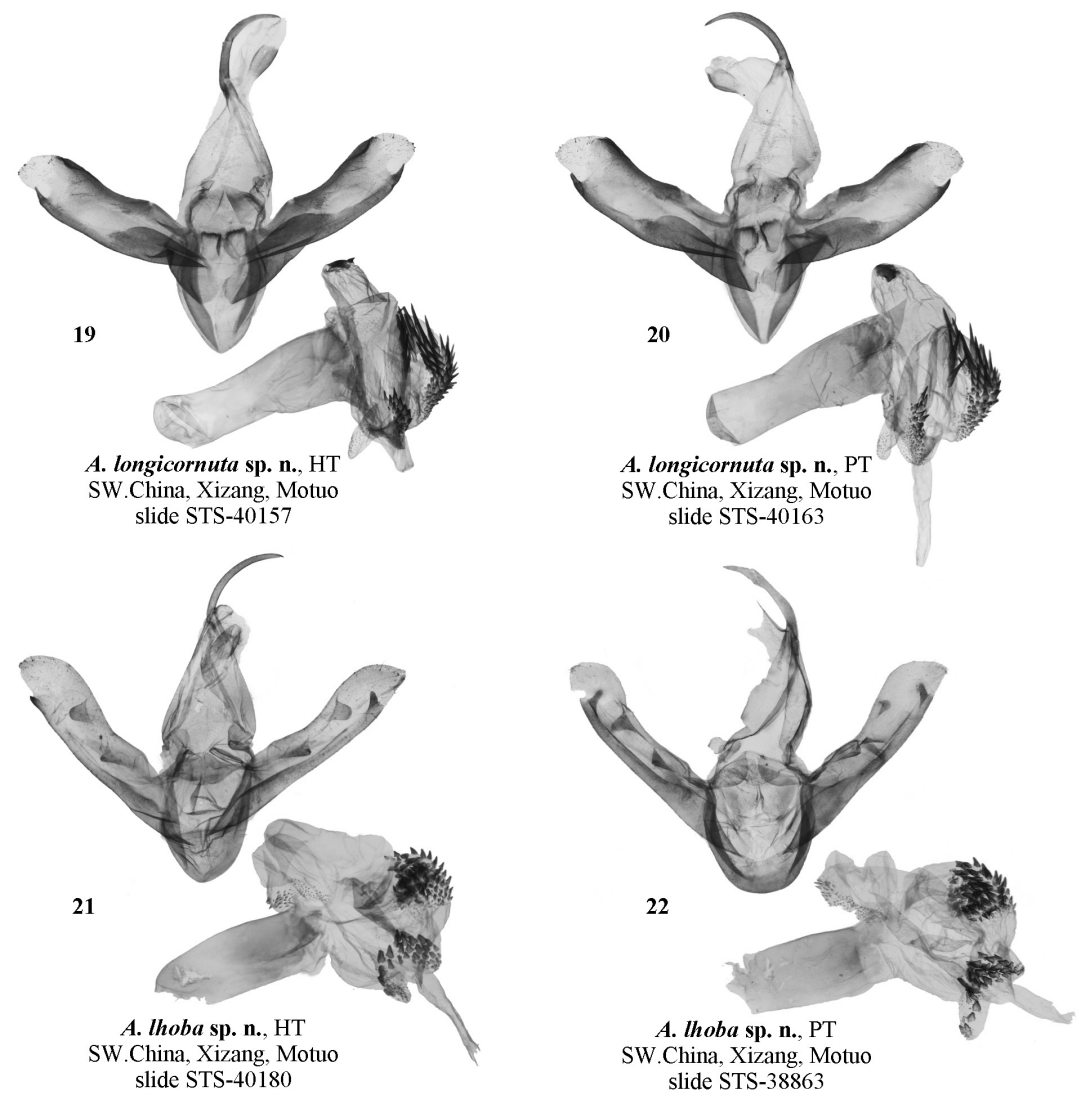
Male genitalia of *Ammathella***19** holotype of *A.longicornuta* sp. nov., slide STS-40157, SW China (TAAHU) **20** paratype of *A.longicornuta* sp. nov., slide STS-40163, SW China (TAAHU) **21** holotype of *A.lhoba* sp. nov., slide STS-40180, SW China (TAAHU) **22** paratype of *A.lhoba* sp. nov., slide STS-38863, SW China (TAAHU).

The female is unknown.

#### Etymology.

The specific epithet *monpa* refers to the local Monpa people in Motuo County.

#### Distribution.

Currently known only from the type locality in Motuo County, southeastern Xizang, China.

### ﻿ Ammathella gesar

Taxon classificationAnimaliaLepidopteraErebidae

(S.-Y. Huang & Volynkin, 2020)

A5FB5B4A-3D5A-5D40-AB44-3BFAC7B27365

Figs 11 , 12 , 25 , 26

Ammatho (Ammathella) gesar S.-Y. Huang & Volynkin in Huang et al., 2020, Zootaxa 4809 (3): 586, figs 7, 8, 19 (type locality: “2130 m, Pailong Village, Linzhi County, Linzhi Division, Xizang Autonomous Region, P. R. China”).

#### Type material examined.

***Holotype***: male, altitude 2130 m, 30.VII.2017, Pailong Village, Linzhi County, Linzhi Division, Xizang Autonomous Region, P. R. China, leg. Si-yao Huang, Shu-qin Ji, Fu-hong Wei and Shi-fang Mo, preparation in glycerol by Huang (SCAU). ***Paratypes***: 2 males, the same data as in the holotype (SCAU).

**Figure 23–26.**
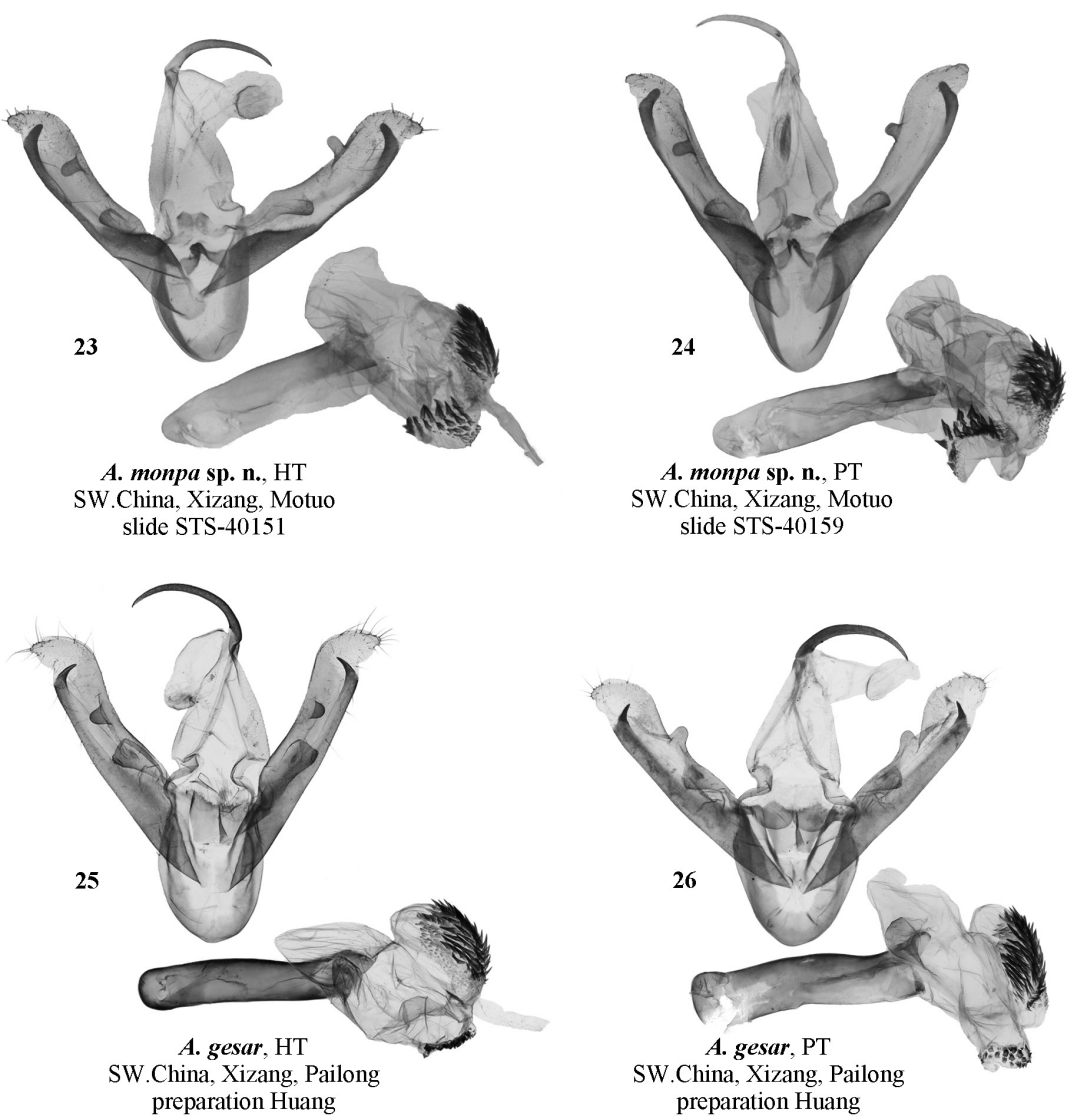
Male genitalia of *Ammathella***23** holotype of *A.monpa* sp. nov., slide STS-40151, SW China (TAAHU) **24** paratype of *A.monpa* sp. nov., slide STS-40159, SW China (TAAHU) **25** holotype of *A.gesar*, SW China (SCAU) **26** paratype of *A.gesar*, SW China (SCAU).

#### Diagnosis.

Length of forewing 11.5–14 mm. *Ammathellagesar* is externally very similar to *A.monpa* sp. nov. and *A.lhoba* sp. nov., and a reliable identification requires the examination of the genitalia structures. Males of *A.gesar* and *A.lhoba* sp. nov. can be distinguished from each other by the distal half of the abdomen, which has a markedly denser admixture of blackish scales in *A.gesar*. The male genitalia of *A.gesar* are most similar to those of *A.monpa* sp. nov., but the genital capsule differs in the longer and broader juxta without a medial process, the shorter distal costal process, and the apically pointed distal saccular process (it is distally thicker and apically rounded in *A.monpa* sp. nov.). Compared to *A.monpa* sp. nov., the phallus of *A.gesar* is slightly narrower, the subbasal diverticulum of the vesica is smaller and distally narrower, and the cornuti on the ventral medial diverticulum are markedly smaller.

The female is unknown.

#### Distribution.

Known only from the type locality in Linzhi County, southeastern Xizang, China.

### ﻿ Ammathella garo

Taxon classificationAnimaliaLepidopteraErebidae

(Volynkin, 2018)

0E0D90D8-1C00-5A9B-B2B2-CB4741A5CF41

**Figure 27–30.**
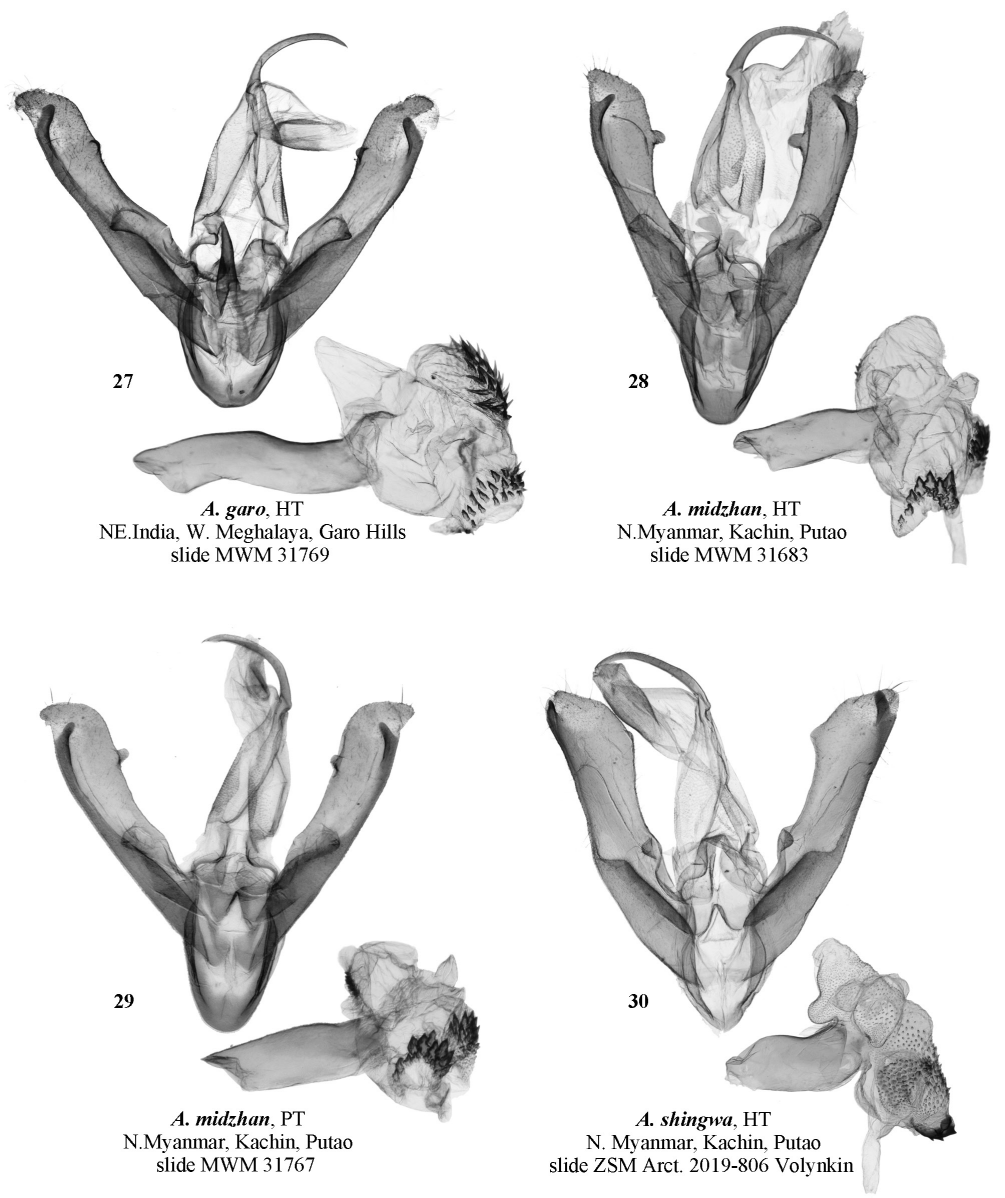
Male genitalia of *Ammathella***27** holotype of *A.garo*, slide MWM 31769, NE India (MWM/ZSM) **28** holotype of *A.midzhan*, slide MWM 31683, N Myanmar (MWM/ZSM) **29** paratype of *A.midzhan*, slide MWM 31767, N Myanmar (MWM/ZSM) **30** holotype of *A.shingwa*, ZSM Arct. 2019–806 Volynkin, N Myanmar (MWM/ZSM).

Figs 13 , 14 , 27 , 33

Barsine garo Volynkin, 2018, Far Eastern Entomologist 358: 6, figs 3, 4, 22, 34 (type locality: “NE India, Assam, W Meghalaya, Garo Hills, Nokrek National Park, 25°40'N, 91°04'E, 1150 m”).

#### Type material examined.

***Holotype***: male, “NE India, Assam, W Meghalaya, Garo Hills, Nokrek National Park, 25°40'N, 91°04'E, 1150 m, 2–13.VII 1997, leg. Afonin & Sinajev [*recte*: Sinyaev]”, slide MWM 31769 Volynkin (MWM/ZSM). ***Paratypes***: 1 male, 6 females, the same data as in the holotype, slide MWM 31770 (female) Volynkin (MWM/ZSM).

#### Diagnosis.

The forewing length is 10–10.5 mm in males and 10–11 mm in females. *Ammathellagaro* is externally similar to *A.midzhan* but differs in the markedly larger size, the more reddish forewing ground color, and the pinkish hindwings. The male genital capsule of *A.garo* is distinguished from that of *A.midzhan* by the narrower tuba analis, the presence of a long conical medial process of the juxta, the somewhat smaller medial costal process, the smaller dorsal subapical costal process, and the more robust distal saccular process. The phallus of *A.garo* is longer (in proportion to the size of the genital capsule) than in *A.midzhan*. The phallus vesica of *A.garo* differs from that of *A.midzhan* in the narrower, conical subbasal diverticulum lacking a cluster of cornuti, and the longer and narrower cornuti of both clusters. In the female genitalia, *A.garo* differs from *A.midzhan* in the larger lateral ostial ligula, the narrower and markedly longer antrum, the weaker sclerotized appendix bursae, and the weaker spinulose scobination of the corpus bursae.

#### Distribution.

Known from northeastern India (Meghalaya) (Volynkin 2018).

### ﻿ Ammathella midzhan

Taxon classificationAnimaliaLepidopteraErebidae

(Volynkin, 2018)

CEEEA093-ED04-5F62-A338-0441AE7C0772

Figs 15 , 16 , 28 , 29 , 34

Barsine midzhan Volynkin, 2018, Far Eastern Entomologist 358: 3, figs 1, 2, 21, 33 (type locality: “Myanmar (Burma), 50 km E Putao, env. Nan Thi village, 950 m”).

#### Type material examined.

***Holotype***: male, “Myanmar (Burma), 50 km E Putao, env. Nan Thi village, 950 m, 17. V. 1998, leg. Murzin & Sinjaev [*recte*: Sinyaev]”, slide MWM 31683 Volynkin (MWM/ZSM). ***Paratypes***: 9 males, 1 female, the same data as in the holotype, slides MWM 31767 (male), MWM 31768 (female) Volynkin (MWM/ZSM).

#### Diagnosis.

The forewing length is 7.5–8 mm in males and 9.5 mm in female. The species is similar to *A.garo*, the detailed comparison is provided above in the diagnosis of the latter species.

#### Distribution.

Known from northern Myanmar (Kachin State).

### ﻿ Ammathella shingwa

Taxon classificationAnimaliaLepidopteraErebidae

(Volynkin & S.-Y. Huang, 2020)

E9AD4AFE-E767-5E81-8DF9-858FFA53C547

Figs 17 , 18 , 30 , 35

Ammatho (Ammathella) shingwa Volynkin & S.-Y. Huang in Huang et al., 2020, Zootaxa 4809 (3): 587, figs 9–10, 20, 25 (type locality: “Myanmar (Burma), 50 km E Putao, env. Nan Thi village, 950 m”).

#### Type material examined.

***Holotype***: male, Myanmar (Burma), 50 km E Putao, env. Nan Thi village, 950 m, 11–16. V.1998, leg. Murzin & Sinjaev, slide ZSM Arct. 2019–806 Volynkin (MWM/ZSM). ***Paratypes***: 1 male, 1 female with the same data as in the holotype, slide ZSM Arct. 2019-821 (male) Volynkin (MWM/ZSM); 1 female, Myanmar (Burma), 50 km NE Putao, Wa Sa Dam village, 950 m, 17.V.1998, leg. Murzin & Sinjaev, slide ZSM Arct. 2019-822 Volynkin (MWM/ZSM).

#### Diagnosis.

The forewing length is 11.5 mm in males and 12.5–13.5 mm in females. *Ammathellashingwa* is externally reminiscent of *A.gesar*, *A.lhoba*, and *A.monpa* but is clearly different by the ochreous abdomen, the markedly narrower reddish pattern elements on the forewing, the thinner postmedial line, and the yellowish hindwing only slightly suffused with pink scales in the submarginal area, whereas in the similar congeners, the abdomen is pinkish with admixture of blackish scales distally, the reddish pattern elements are broad, and the hindwing is pinkish. The male genitalia of *A.shingwa* are most similar to those of *A.midzhan* and *A.lhoba* due to sharing similar short phallus. However, the species can easily be distinguished from *A.midzhan* and the externally similar congeners by the distally dilated valva with a longer and broader trapezoid basal section of the sacculus and the vesica lacking a medial semiglobular diverticulum with a cluster of cornuti and thoroughly covered with granulation. The female genitalia of *A.shingwa* differ from those of the congeners in the broader antrum, the broader corpus bursae with a smaller signum, and the larger and more heavily sclerotised appendix bursae.

#### Distribution.

Known from northern Myanmar (Kachin State).

**Figure. 31–35.**
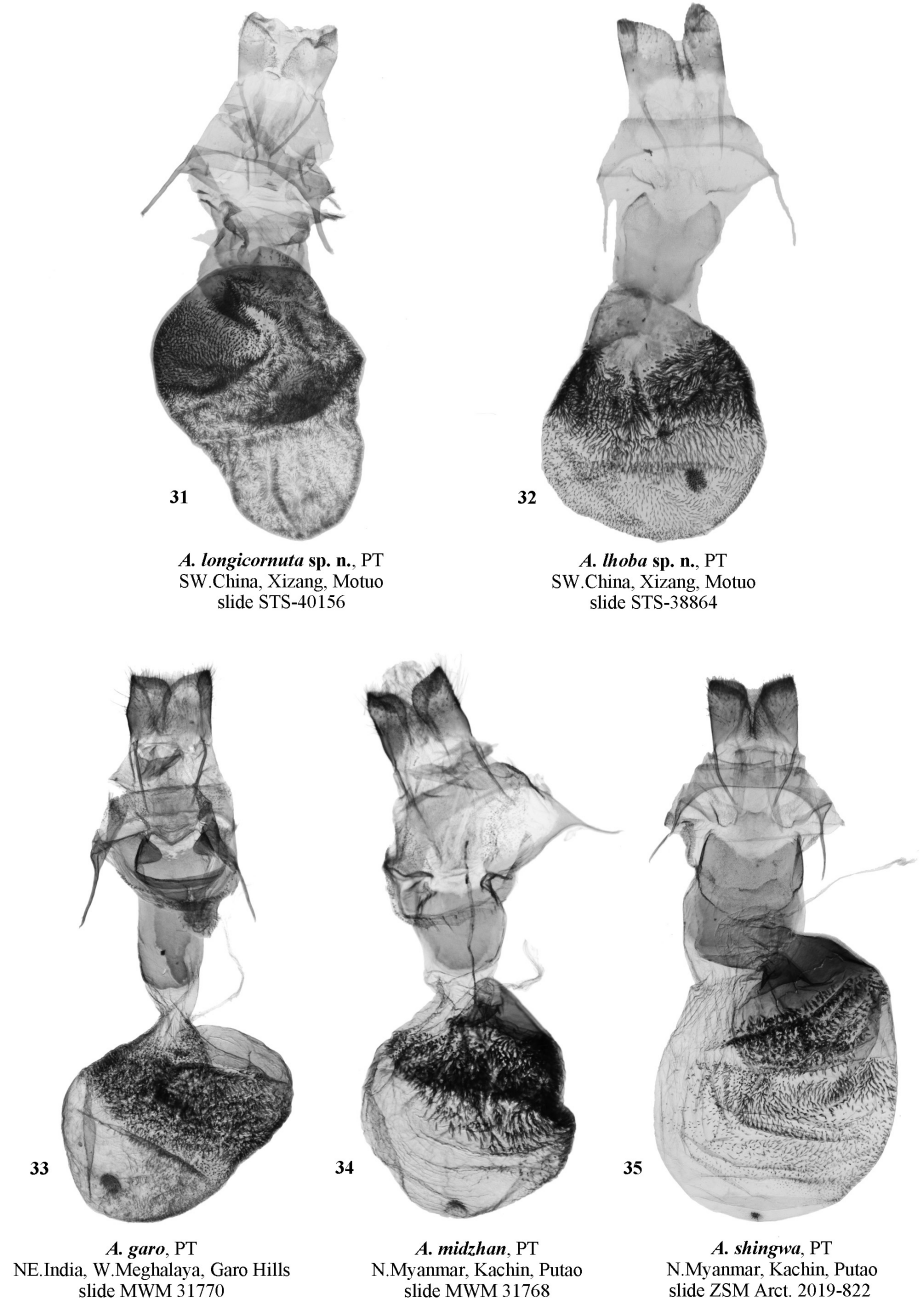
Female genitalia of *Ammathella***31** paratype of *A.longicornuta* sp. nov., slide STS-40156, SW China (TAAHU) **32** paratype of *A.lhoba* sp. nov., slide STS-38864, SW China (TAAHU) **33** paratype of *A.garo*, slide MWM 31770, NE India (MWM/ZSM) **34** paratype of *A.midzhan*, slide MWM 31768, N Myanmar (MWM/ZSM) **35** paratype of *A.shingwa*, ZSM Arct. 2019–822 Volynkin, N Myanmar (MWM/ZSM).

### ﻿Key to the species of the genus *Ammathella* based on morphology of males

**Table:** 

1	Tip of abdomen mainly covered with blackish or darkish hair-like scales	**4**
–	Tip of abdomen lacking admixture of blackish or darkish hair-like scales	**2**
2	Hindwing reddish; semiglobular medial diverticulum in phallus vesica scattered with long and large cornuti basally	***A.longicornuta* sp. nov.**
–	Hindwing pale pinkish or yellowish; semiglobular medial diverticulum in phallus vesica absent or (if present) covered with short and robust cornuti thoroughly	**3**
3	Hindwing pale pinkish; semiglobular medial diverticulum in phallus vesica present, covered with short and robust cornuti thoroughly	***A.lhoba* sp. nov.**
–	Hindwing pale yellowish; semiglobular medial diverticulum in phallus vesica absent, and ventral medial diverticulum bearing a cluster of short and robust cornuti	***A.shingwa***
4	Juxta with a medial process	**5**
–	Juxta without a medial process	**6**
5	Juxta with a long conical process, subbasal diverticulum conical	***A.garo***
–	Juxta with a short and rounded process covered with spinules, subbasal diverticulum sack- like	***A.monpa* sp. nov.**
6	Distal saccular process apically pointed; phallus elongate; semi-globular medial diverticulum covered with long, spine-like cornuti	***A.gesar***
–	Distal saccular process apically rounded; phallus short and stout; semi-globular medial diverticulum covered with short and robust cornuti	***A.midzhan***

## Supplementary Material

XML Treatment for Ammathella

XML Treatment for Ammathella longicornuta

XML Treatment for Ammathella lhoba

XML Treatment for Ammathella monpa

XML Treatment for Ammathella gesar

XML Treatment for Ammathella garo

XML Treatment for Ammathella midzhan

XML Treatment for Ammathella shingwa

## References

[B1] Huang SY Volynkin AV Wang M Fan XL (2020) Three new species of the genus *Ammatho* Walker, 1855 from China and Indochina (Lepidoptera: Erebidae: Arctiinae: Lithosiini). Zootaxa 4809(3): 582–592. 10.11646/zootaxa.4809.3.11 33055931

[B2] Huang SY Volynkin AV Miao ZP Tan SY Wang M Fan XL (2021) Molecular phylogeny and classification of Nudariina (Lepidoptera: Erebidae) with emphasis on the genera *Barsine* Walker, *Ammatho* Walker and *Ovipennis* Hampson. Systematic Entomology 46(4): 1045–1059. 10.1111/syen.12509

[B3] Volynkin AV (2018) Four new species of the genus *Barsine* Walker, 1854 (Lepidoptera, Erebidae, Arctiinae) from Oriental Region. Far Eastern Entomologist 358: 1–18. 10.25221/fee.358.1

[B4] Volynkin AV Huang SY Ivanova MS (2019) An overview of genera and subgenera of the *Asura* / *Miltochrista* generic complex (Lepidoptera, Erebidae, Arctiinae). Part 1. *Barsine* Walker, 1854 sensu lato, *Asura* Walker, 1854 and related genera, with descriptions of twenty new genera, ten new subgenera and a check list of taxa of the *Asura/Miltochrista* generic complex. Ecologica Montenegrina 26: 14–92. 10.37828/em.2019.26.3

[B5] Walker F (1854) List of the Specimens of Lepidopterous Insects in the Collection of the British Museum. Part I. – LepidopteraHeterocera. The Trustees of the British Museum, London, 278 pp. 10.5962/bhl.title.58221

[B6] Walker F (1855) List of the specimens of lepidopterous insects in the collection of the British Museum. Vol. 3. Printed by order of the Trustees, London, 583–775.

